# Graphene Oxide and Its Inorganic Composites: Fabrication and Electrorheological Response

**DOI:** 10.3390/ma12132185

**Published:** 2019-07-07

**Authors:** Yu Zhen Dong, Joo Nyeon Kim, Hyoung Jin Choi

**Affiliations:** Department of Polymer Science and Engineering, Inha University, Incheon 22212, Korea

**Keywords:** graphene oxide, inorganic, composite, electrorheological

## Abstract

Composite particles associated with graphene oxide (GO) and inorganic materials provide the synergistic properties of an appropriate electrical conductivity of GO with the good dielectric characteristics of inorganic materials, making them attractive candidates for electrorheological (ER) materials. This review paper focuses on the fabrication mechanisms of GO/inorganic composites and their ER response when suspended in a non-conducting medium, including steady shear flow curves, dynamic yield stress, On-Off tests, and dynamic oscillation analysis. Furthermore, the morphologies of these composites, dielectric properties, and sedimentation of the ER fluids are covered.

## 1. Introduction

Graphene, a 2-D carbon sheet comprised of sp^2^ hybridized carbon atoms packed in a regular atomic-scale hexagonal structure, has attracted considerable attention in many fields because of its special physical and chemical properties, such as high specific surface area, high flexibility and electric conductivity, enhanced thermal stability, and good mechanical properties. Based on these excellent properties, graphene-based materials have potential applications in sensors, catalysts, adsorbents, capacitors, and electromagnetic interference (EMI) shielding devices, etc. [1,2,3,4,5,6]. On the other hand, the preparation of a high quality graphene sheet is still challenging. Compared to graphene, the process for preparing graphene oxide (GO) using graphite is becoming mature with a low cost and easy application to industrial mass production [7,8]. GO is the oxidized form of graphene, which not only retains most of the excellent properties of graphene, but contains numerous oxygen functional groups on the surface that enhance its dispersion stability in aqueous and organic solvents. Although GO has many unique properties, its poor electrical conductivity compared to graphene has limited applications in many electrochemical fields. Therefore, reduced GO (rGO) has been proposed as a solution for improving the electrical conductivity of GO, and avoiding the difficulty of graphene production, which can be produced by the reduction of GO using chemical and thermal methods [9,10]. Therefore, both rGO and GO-based materials have attracted considerable interest in many fields [11,12,13,14].

Smart materials are designed materials with one or more stimuli-response properties and form the basis for many industrial applications. The properties of such materials can vary significantly due to input stimuli, including temperature, light, electric field, magnetic field, mechanical, and chemicals [15,16,17,18]. In particular, both electrorheological (ER) and magnetorheological (MR) suspensions are very important intelligent materials, whose rheological characteristics could be altered by the presence of an electrical and magnetic field, respectively [19,20,21,22]. In particular, ER fluids usually consisted of semi-conducting or polarizable materials suspended in insulating media, and their rheological properties can be tuned by electrical field stimuli. In the absence of an electrical field, ER suspensions exhibit Newtonian liquid-like behavior, but under an applied electric field, their rheological properties show Bingham plastic behavior. When the applied electrical field is removed, the ER suspension returns to the original Newtonian fluid-like phase. Owing to these reversible characteristics, ER fluids have been used widely in many fields in industry, such as damper systems, actuators, brakes, robotics, and clutches [23,24,25,26,27].

The response of ER fluids under electrical field stimulation has been attributed to the polarization behavior of the suspended particles compared to the dispersion medium caused by an external electric field. This polarization may come from a variety of charge transport mechanisms, including electrons, dipoles, atoms, or interfacial polarization. The connected polarized particles attract each other when they are paired in the direction parallel to the electric field, and repel each other in the vertical electrical field direction, thereby causing them to form a chain structure in the parallel electrical field direction. Therefore, under an electrical field, to make particles move with the flow, the hydrodynamic force must act on the electrostatic force between the particles to deform or even destroy the fibrous structure, leading to an increase in shear stress and shear viscosity. Based on the electrostatic polarization mechanism, many models have been proposed to evaluate the magnitude of the electrostatic force caused by an electric field. In idealized electrostatic polarization theory, it is envisaged that ER fluids are composed of suspended, hard, monodisperse spherical particles suspended in a Newtonian liquid, and both phases are assumed to be uncharged and non-conducting. In this model, the mismatch of permittivity between the dispersed phase and continuous phase is considered to be the cause of the electrostatic forces. The electrostatic interactions among particles can be expressed as $F\approx {4\pi a}^{2}\epsilon_{0}\epsilon_{c}E^{2}{\left(a/\delta \right)}^{2}$, where $\epsilon_{0}$ and $\epsilon_{c}$ are the permittivity of a vacuum and continuous phase, respectively; a is the radius of the particles, and δ represents the radius of the inner region of neighbor particles. Furthermore, considering only the electrostatic interactions of neighboring particles, the dynamic yield stress can be expressed as $\tau_{0}=18\phi \epsilon_{0}\epsilon_{c}E_{0}^{2}f_{m}\left[1-\frac{{\left(\pi /6\right)}^{1/2}}{\left(L/a\right){\tan\theta }_{m}\phi^{1/2}}\right]$, where ϕ is the volume fraction of the ER fluid; $f_{m}\left(\alpha \right)$ is a maximum in the dimensionless restoring force; $\theta_{m}\left(\alpha \right)$ is the maximum angle between the direction of the chain and direction of the electric field [28,29,30,31].

For decades, numerous particles have been introduced as ER materials, such as polyaniline (PANI), polypyrrole (PPy), polydiphenylamine (PDPA), polyindole (PIn), clay, and oxide mineral [32,33,34,35,36,37,38,39]. Recently, graphene, rGO and GO-based materials have attracted considerable attentions as candidates of ER materials. Dhar et al. [40] reported large effects in polyethylene glycol (PEG 400)-based graphene gels. Dong et al. [41] fabricated a graphene/Mg-Al layered double-hydroxide (LDH) composite for an ER material, where the LDH provided an electrical insulating effect for graphene. Yin et al. [42] developed a core-shell structured rGO-PPy and reported enhanced ER effects compared to PPy. High electrical conductivity can easily produce a high leakage current; therefore, compared to graphene and rGO, GO has attracted increasing attention as an ER material. Pure GO was adopted as an ER particle, owing to its relatively low electrical conductivity. Zhang et al. [43] synthesized GO and dispersed it in silicone oil to prepare a GO-based ER fluid that exhibited typical ER properties, whereas the ER performance was relatively low. Hong et al. [44] applied an efficient dispersion technique of solvent exchange to prepare a GO-based ER fluid and observed an enhanced ER effect. Shin et al. [45] synthesized GO sheets with a uniform sub-micrometer size using a mechano-chemical process, and the density of GO was decreased using a ball-milling process. The uniform size and low density contributed to the better dispersion stability of GO in silicone oil and was beneficial in obtaining stronger ER effects. Moreover, many GO-based composites have also been developed, including GO/polymer and GO/inorganic composites [46,47,48,49,50].

Layered graphene has delocalized π bonds formed by sp^2^ hybridized orbitals between layers, in which electrons can move freely, so that graphene has a very strong electrical conductivity. GO is an oxidized state of graphene, resulting in destruction of the conjugated structure to be reduced conductivity. On the other hand, electron transport between the layers can also occur in GO because graphene usually is not oxidized completely and is generally not peeled off into a single-layer structure. In addition, the oxygen-containing groups on the surface of the GO give it greater polarizability.

The polar functional groups on the surface of the GO, as well as the inorganic components, allow GO/inorganic materials to be polarized by an external electric field, and the insertion of inorganic components makes the polarizability of GO/inorganic composites higher than GO, which gives the composites a chance to have higher ER characteristics. Therefore, the transport of electrons between the GO layers, the polarization of polarizable functional groups, and the polarization of inorganic components contribute to the ER effect of the GO/inorganic composites.

In particular, composites of GO and inorganic materials, such as Al_2_O_3_, TiO_2_, SiO_2_, and Fe_3_O_4_, have attracted considerable attention because they can combine the appropriate conductivity of GO with the good dielectric properties of inorganic materials. Because the dielectric characteristics are strongly associated with the ER effects, the insertion of high dielectric constant inorganic substances can improve the achievable interfacial polarization of GO/inorganic properties, resulting in improved ER characteristics. This article reviews the fabrication mechanisms of various GO/inorganic composite particles and their ER properties. Their dielectric properties are also related to corresponding ER performance.

## 2. Fabrication and Morphologies

### 2.1. Electrostatic Interaction

Because GO sheets possess many oxygen functional groups, including epoxy, hydroxyl, and carboxyl groups, GO generally exhibits a strong negatively charged property. Therefore, synthetic strategies utilizing electrostatic interactions are used widely as a facile method for the synthesis of GO-based composites. To synthesize composites with negatively charged GO through an electrostatic interaction, the other component needs to be positively charged. A few different methods can be used to realize the electrostatic interactions between GO and inorganic particles.

#### 2.1.1. Adjustment of the pH

In an aqueous solution environment, for a mineral oxide, water can be adsorbed chemically onto the oxide by hydrogen bonding, or it can be dissociated. [51] The dissociation process can induce the formation of surface hydroxyl groups, which exhibit amphoteric character and may determine the surface charge as follows:

(1) $${S-\mathrm{OH}}_{2}^{+}\;⇌{\;S-\mathrm{OH}\;+\;H}^{+}$$

(2) $$S-\mathrm{OH}\;⇋{\;S-O}^{-}{+\;H}^{+}$$

**Figure 1:**
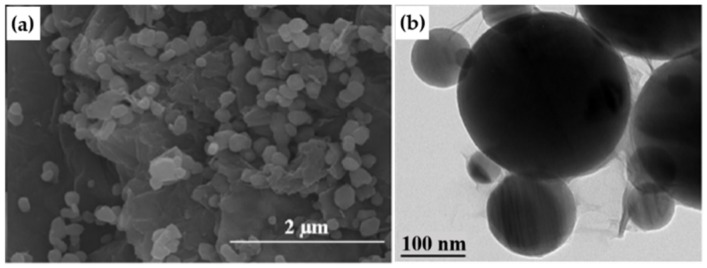
(**a**) SEM image of GO/TiO_2_ [53], (**b**) TEM image of GO wrapped Al_2_O_3_ [54].

Therefore, the surface charge of oxide particles can be controlled by adjusting the pH [52]. Zhang et al. [53] obtained positively charged titanium dioxide (TiO_2_) by treating TiO_2_ nanoparticles with a 1M HCl solution and GO/TiO_2_ composites were prepared by electrostatic interactions between negatively charged GO and positively charged TiO_2_. As shown in the scanning electron microscopy (SEM) image (Figure 1a), nanosized TiO_2_ particles were loaded successfully on the GO sheet. The GO wrapped alumina (Al_2_O_3_) prepared by Zhang et al. [54] also employed the same method to adjust the surface charge of the spherical Al_2_O_3_ particles, and the GO sheet then covered the Al_2_O_3_ particle through electrostatic attraction, which was confirmed by transmission electron microscopy (TEM) (Figure 1b).

#### 2.1.2. Ultrasonic-Assisted Synthesis

**Scheme 1:**
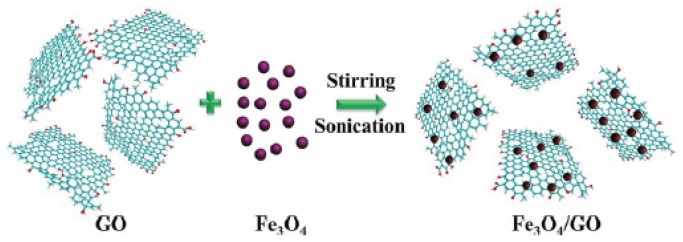
Schematic diagram of the preparation process of Fe_3_O_4_/GO [55].

Zhang et al. [55] synthesized a Fe_3_O_4_/GO composite using an ultrasonic-assisted electrostatic reaction synthetic route and adopted it as an electro-magneto dual stimuli-responsive material. Scheme 1 presents the preparation process. The GO sheets and Fe_3_O_4_ nanoparticles were prepared using the modified Hummers’ method and a chemical co-precipitation method, respectively. Subsequently, Fe_3_O_4_ nanoparticles were added to the aqueous suspension of GO, and the pH of the suspension of GO was adjusted to 4 to impart a positive charge to the Fe_3_O_4_, and the Fe_3_O_4_/GO composite was then synthesized by an electrostatic reaction assisted by sonication. The Fe_3_O_4_/GO composite combines the paramagnetic properties of Fe_3_O_4_ nanoparticles with the polarization properties of GO. Therefore, their suspension exhibited excellent electro-magneto dual stimuli-responsive behaviors.

#### 2.1.3. Surface Modification

**Figure 2:**
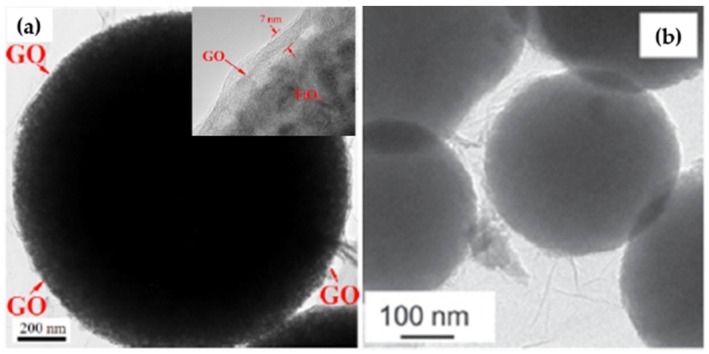
TEM images of (**a**) GO-wrapped TiO_2_ microspheres [56] and (**b**) core-shell structured TiO_2_/GO [58].

**Scheme 2:**
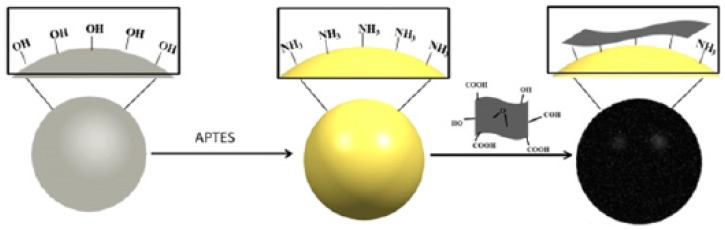
Synthetic pathway of core-shell typed TiO_2_/GO [58].

The grafting of a positively charged group onto particles is another method. Yin et al. [56] prepared amine-terminated TiO_2_ by dispersing it in an ethanol solution of 3-aminopropyl-triethoxy-silane (APTES) using a silanization reaction between the surface hydroxyl groups of TiO_2_ and ethoxy groups of APTES [57]. GO-wrapped TiO_2_ microspheres were then prepared by coating GO on the positively charged amine-terminated TiO_2_ using an electrostatic interaction. The diameter of GO-wrapped TiO_2_ microspheres was 800–1000 nm and the thickness of GO was approximately 5–10 nm (Figure 2a). The ER fluid based on GO-wrapped TiO_2_ microspheres showed better stability, as well as higher yield stress and storage modulus than the bare TiO_2_ microspheres because the GO layer on the TiO_2_ enhances the interfacial polarizability. Dong et al. [58] fabricated core-shell structured TiO_2_/GO using the same mechanism. Scheme 2 shows the synthetic pathway of core-shell typed TiO_2_/GO. The size distribution of the particles ranged from 400 to 500 nm, and a thin GO layer was observed at the margin of TiO_2_ in the TEM image (Figure 2b). They also synthesized TiO_2_/urea and adopted it as an ER material. Both TiO_2_/urea and TiO_2_/GO-based ER suspensions showed better ER effects than bare TiO_2_ because of the polar groups of urea and GO. In addition, TiO_2_/GO-based ER fluid even showed enhanced yield stress, reduced leakage current density, and improved sedimentation behavior compared to TiO_2_/urea, which may be because the GO layer not only provides more polar groups, but also increases the interaction areas between the particles.

**Figure 3:**
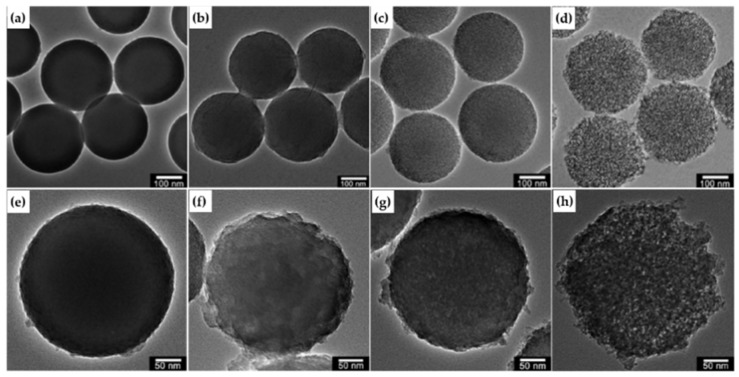
TEM images of (**a**) nSiO_2_, (**b**) p1SiO_2_, (**c**) p2SiO_2_, (**d**) epSiO_2_, (**e**) GO/nSiO_2_, (**f**) GO/p1SiO_2_, (**g**) GO/p2SiO_2_, and (**h**) GO/epSiO_2_. [59].

Yoon et al. [59] fabricated several density-controlled GO coated-mesoporous SiO_2_ spheres using silica with different pore sizes through an amine-modification route similar to TiO_2_/GO. Figure 3a–d present TEM images of synthesized nSiO_2_, p1SiO_2_, p2SiO_2_, and epSiO_2_ with a pore size of 0, 1.10, 2.21, and 10.24 nm, respectively. They exhibited spherical morphologies with a diameter of approximately 270 nm. Figure 3e–h show TEM images of the GO-coated silica spheres (GO/nSiO_2_, GO/p1SiO_2_, GO/p2SiO_2_, and GO/epSiO_2_), in which the thickness of GO layers was less than 10 nm, whereas the densities of GO/nSiO_2_, GO/p1SiO_2_, GO/p2SiO_2_, and GO/epSiO_2_ were determined to be 2.67, 2.52, 2.28, and 2.03 g/cm^3^, respectively. Among them, the GO/epSiO_2_-based ER suspension showed the highest yield stress, largest polarizability, and shortest relaxation time of interfacial polarization. The improved ER performance was attributed to the reduced particle density and altered dielectric properties.

**Figure 4:**
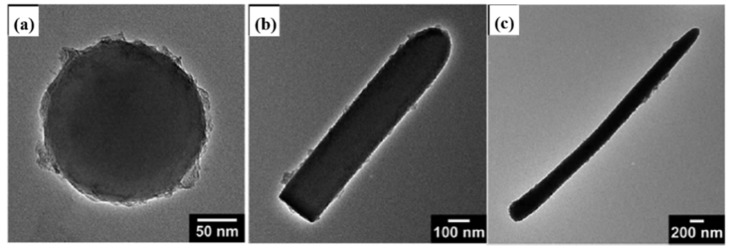
TEM images of GO-wrapped silica sphere (**a**) and GO-wrapped silica spheres rod with an aspect ratio of 5.0 (**b**), rod with an aspect ratio of 20 (**c**) [60].

**Figure 5:**
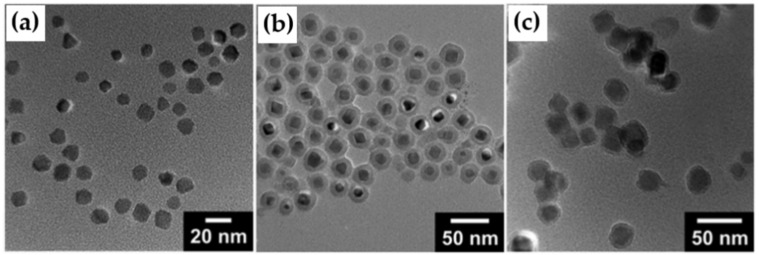
TEM images (**a**) Fe_3_O_4,_ (**b**) Fe_3_O_4_/SiO_2,_ and (**c**) Fe_3_O_4_/SiO_2_/GO [65].

Lee et al. [60] synthesized silica with different aspect ratios, including SiO_2_ spheres with a diameter of 200 nm and SiO_2_ rods with aspect ratios of 1.8, 3.1, 5.0, 8.0, and 20. These were then modified with amine groups and coated with GO to form GO-coated silica with different aspect ratios. Figure 4a presents TEM images of GO-coated silica spheres, whereas Figure 4b,c show GO-coated silica rods with aspect ratios of 5.0 and 20, respectively. The relationship between the aspect ratio of the GO-coated silica particles and ER effect was investigated. The ER performance of ER particles increased with an increasing aspect ratio. The enhancement of the ER performance can be explained by the following: (1) the particle with high aspect ratio increases the contact area between particles and enhances the resistance; and (2) the material with a higher aspect ratio provides increased polarizability and a shorter relaxation time of interfacial polarization. The influence of geometrical morphologies on the ER characteristics has always been an important topic. Many studies have shown that rod-shaped, tubular, and fibrous ER materials usually exhibit a stronger ER effect than granular materials [61,62,63]. In addition, ER materials with a high aspect ratio can exhibit better sedimentation stability [64]. Core-shell structured GO-attached iron oxide/silica nanoparticles (Fe_3_O_4_/SiO_2_/GO) [65] were also prepared by an electrostatic reaction and used as electro-magneto dual stimuli-responsive material. Figure 5 presents TEM images of Fe_3_O_4_, Fe_3_O_4_/SiO_2_, and Fe_3_O_4_/SiO_2_/GO. The diameter of the Fe_3_O_4_, Fe_3_O_4_/SiO_2_, and Fe_3_O_4_/SiO_2_/GO nanoparticles were 12, 24, and 28 nm, respectively, showing that the thickness of the GO layer is approximately 2.0 nm.

### 2.2. In-situ Growth

**Figure 6:**
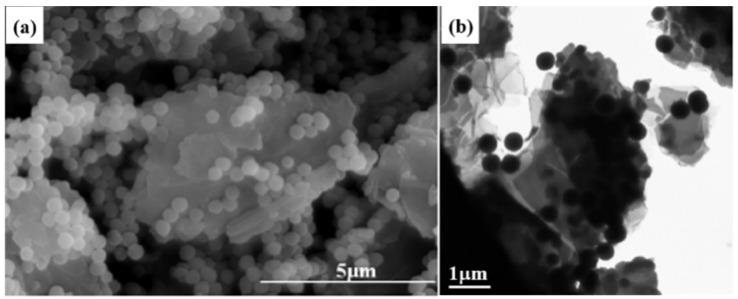
(**a**) SEM and (**b**) TEM images of Si-GO [66].

Zhang et al. [66] added tetraethyl orthosilicate (TEOS) as a silicon source reagent to the suspension of GO with hydrous ammonia (NH_3_·H_2_O) and fabricated a Si-GO composite using the in-situ hydrolysis of TEOS on the GO sheet. Because the in-situ growth method utilizes a covalent bond between silica (Si-O-C) and GO (C=O), the composite prepared by this method generally has better stability. SEM and TEM images of the Si-GO (Figure 6) showed that the nanosized silica spheres had been attached successfully to the surface of GO sheets. Li et al. [67] used an amphiphilic copolymer ((PEO) 106 (PPO) 70 (PEO) 106) to promote the heterogeneous nucleation of TEOS on a GO sheet and obtained GO/SiO_2_ by in-situ hydrolysis. They also obtained r-GO/SiO_2_ by reducing GO/SiO_2_ in a hydrazine solution and evaluated its dielectric properties and ER effects.

### 2.3. Chemical Grafting

**Scheme 3:**
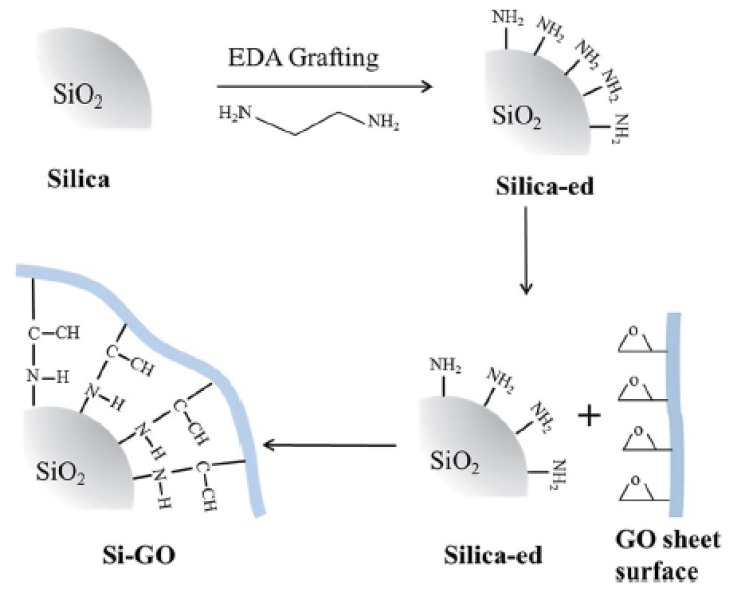
Schematic diagram of the synthetic route of GO-coated silica [68].

**Figure 7:**
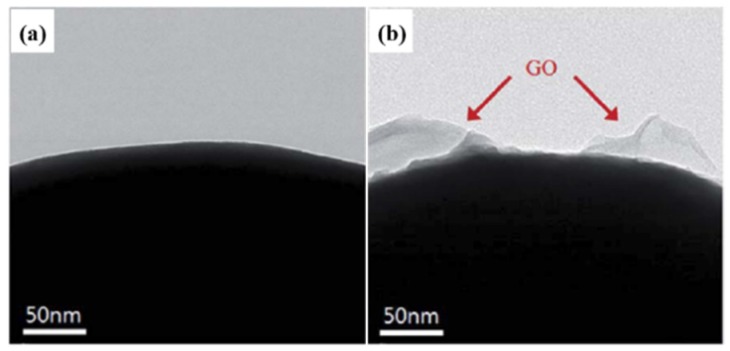
TEM images of (**a**) silica and (**b**) GO coated silica [68].

GO has high chemical reactivity owing to the abundant functional groups on its surface, making it is possible to assemble GO composites based on a range of specific chemical reactions. Kim et al. [68] fabricated core-shell-structured GO coated silica particles by grafting ethanediamine (EDA) on silica and coating a GO shell using the epoxy-amine reaction between the amine groups and epoxy groups on the GO surface. Scheme 3 shows a schematic diagram of the synthetic route. The TEM image of GO-coated silica (Figure 7b) revealed a distinct difference from silica (Figure 7a), with a rugged GO coating on the edge. Although the chemical grafting method appeared to be similar to the surface modification method mentioned above, chemical grafting undergoes a chemical reaction, whereas surface modification aims to synthesize the composite using mainly electrostatic interactions. Therefore, the connection between the components of the composite material prepared by the chemical grafting method could be stronger. 

Overall, all of these methods can synthesize GO/inorganic composite materials. Among them, the method of fabrication by electrostatic interactions is generally simple in operation and cost. On the other hand, the in-situ growth or chemical grafting method, although requiring relatively fast reaction conditions and complicated operation, may contribute to a stronger binding force and more stable composites because they can generate chemical bonds between the GO and inorganic substances. From the perspective of economy and ease of operation, it is believed that for general GO/inorganic composites, synthetic methods using electrostatic interactions are the most highly recommended.

## 3. Electrorheological Characteristics

### 3.1. Formation of Chain-Like Structures

**Figure 8:**
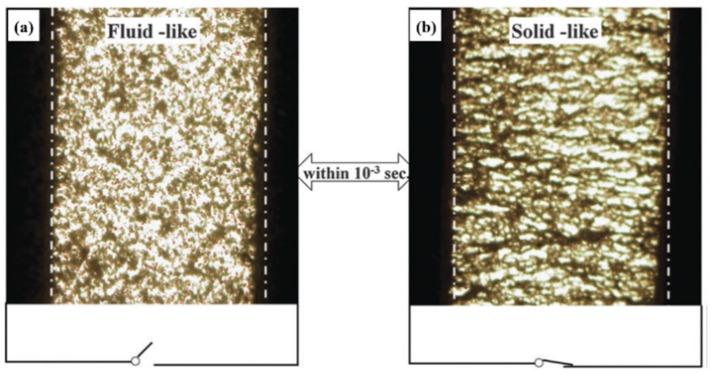
Optical microscopy images of GO/TiO_2_-based ER fluid in the absence of an electric field (**a**) and under an applied electric field (**b**) [53].

ER fluids are typically composed of polarizable solid particles dispersed in an insulating carrier liquid. The state of the ER fluids is tunable between fluid-like and solid-like within milliseconds by the application or removal of an external electrical field. Figure 8 presents the microstructural changes to the GO/TiO_2_ suspended in silicone oil (15 wt.%) under a DC electrical field. The ER fluid in the absence of an electrical field indicates that the particles are freely suspended in silicone oil, at which point, the ER suspension exhibits general fluid-like characteristics. On the other hand, in the presence of an electrical field, the particles will be polarized and arranged in a chain-like structure along the electric field direction in an extremely short time, resulting from electrostatic interactions between polarizable particles. At this time, the ER suspension exhibits solid-like properties, usually expressed as instantaneously enhanced shear stress, shear viscosity, and dynamic moduli [69]. In the following, the related behaviors of the ER fluid will be introduced individually.

### 3.2. Dielectric Properties

The characteristics of ER suspensions under an electrical field are controlled mainly by the competition of electrostatic interactions caused by an electrical field and the hydrodynamic interactions from fluid flow. Therefore, the dielectric characteristics are considered to be strongly associated with the ER performance.

Figure 8 shows the dielectric properties of various ER fluids; the fitted lines are generated from the following Cole-Cole equation [70]:

(3) $$\varepsilon *=\varepsilon^{\prime }+i\varepsilon^{\prime \prime }=\varepsilon_{\infty }+\frac{\varepsilon_{0}-\varepsilon_{\infty }}{1+{\left(i\omega \lambda \right)}^{1-\alpha }};\;0\;\leq \;\alpha \;<\;1$$

According to this equation, the permittivity ($\varepsilon^{\prime }$) and loss factor ($\varepsilon^{\prime \prime }$) can be expressed as

(4) $${\varepsilon^{\prime }\;=\;\varepsilon }_{\infty }+\left(\varepsilon_{0}-\varepsilon_{\infty }\right)\frac{1+(\omega \lambda {)}^{1-\alpha }\cos\frac{\pi (1-\alpha )}{2}}{1+2(\omega \lambda {)}^{1-\alpha }\cos\frac{\pi (1-\alpha )}{2}{+(\omega \lambda )}^{2(1-\alpha )}}$$

(5) $$\varepsilon^{\prime \prime }\;=\;\frac{(\varepsilon_{0}-\varepsilon_{\infty })(\omega \lambda {)}^{1-\alpha }\sin\frac{\pi (1-\alpha )}{2}}{1+2(\omega \lambda {)}^{1-\alpha }\sin\frac{\alpha \pi }{2}+(\omega \lambda {)}^{2(1-\alpha )}}$$

Here $\varepsilon^{*}$ is the complex dielectric constant; $\varepsilon^{\prime }$ and $\varepsilon^{\prime \prime }$ are the permittivity and loss factor, respectively; $\varepsilon_{0}$ and $\varepsilon_{\infty }$ are the permittivity as the frequency approaches zero and infinity, respectively; $\Delta \varepsilon =\varepsilon_{0}-\varepsilon_{\infty }$; ω = 2πf, where f is the frequency; λ represents the interfacial polarization rate, $\lambda =1/(2\pi f_{\max})$, where $f_{\max}$ is the frequency at which the loss factor reaches its maximum. The exponent α is a value between 0 and 1, which describes the different polarization relaxation situation.

**Figure 9:**
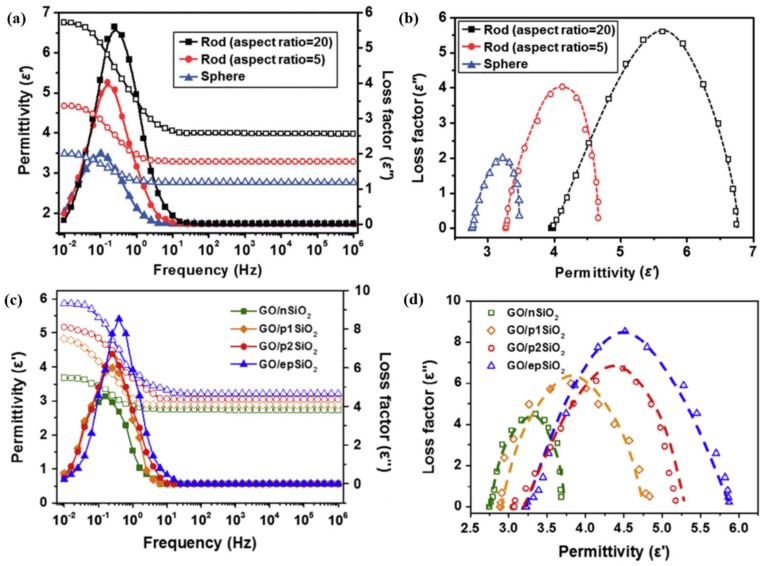
Dielectric properties of ER fluids based on (**a**,**b**) GO-wrapped silica with different aspect ratios [59] and (**c**,**d**) GO/SiO_2_ spheres with different densities [59].

Δε is associated with the degree of achievable polarization of ER fluids, and the degree of polarization that can be achieved increases with increasing Δε. The λ reflects the relaxation time of interfacial polarization. When λ is too high, the ER particles are unable to achieve sufficient polarization in time and the chain-like structures cannot be recombined in a timely manner in flow. On the other hand, a too low λ value might cause repulsive interactions between the particles because of the different polarization direction of the neighbor particles. The ER fluid with a $f_{\max}$ ranging from 10^2^ to 10^5^ Hz, i.e., λ ranging from approximately 10^−6^ to 10^−3^ s, may contribute to stable flow behavior under a steady-shear flow. Figure 9a,b demonstrates the dielectric behaviors of ER suspensions based on a series of GO-wrapped silica, and fitted by the Cole-Cole equation. The Δε values for ER fluids based on GO-attached silica spheres, rods with an aspect ratio of 5, and rods with an aspect ratio of 20 were 0.71, 1.40, and 2.78, respectively. In addition, their λ values were 1.592, 1.007, and 0.634, respectively. This shows that an ER fluid based on GO-attached silica rods with the highest aspect ratio showed the highest achievable polarizability and fastest polarization rate, resulting in the highest ER effect, which is consistent with the conclusions inferred from the dielectric properties. The ER fluids based on GO/SiO_2_ spheres with different densities were produced. Figure 9c,d also showed different dielectric properties, while also exhibiting a range of ER properties. The Δε for ER fluids based on GO/SiO_2_ spheres with a density of 2.67, 2.52, 2.28, and 2.03 g/cm^3^ were 0.94, 1.94, 2.12, and 2.65, respectively. In addition, their λ values were 1.00, 0.64, 0.64, and 0.40 s, respectively, suggesting that particles with a low density may contribute to high achievable polarization and short relaxation time, thereby contributing to achieving a high ER effect. In their study, they confirmed that ER fluids based on low-density particles exhibit better ER effects. In addition, unstable stress regions can be observed in their shear stress curves, which may be related to the relatively higher relaxation times. This is because a higher relaxation time may result in the particles not being able to achieve sufficient polarization rapidly in the flow field, resulting in the chain structure not being reorganized in time.

### 3.3. Steady-Shear Flow Curves

**Figure 10:**
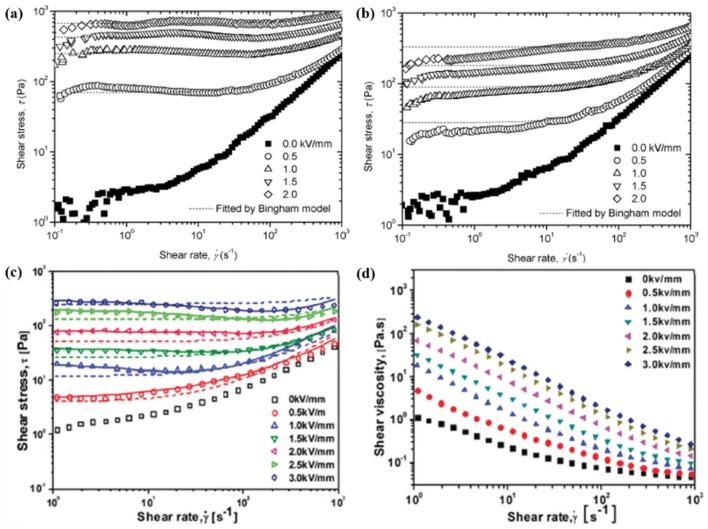
Shear stress of GO/SiO_2_ [67] based ER fluid (3 vol.%) under (**a**) DC electric fields and (**b**) AC electric fields (1 kHz), and (**c**) shear stress, (**d**) shear viscosity curves of Si-GO based ER fluid (9 wt.%) [66].

In steady shear tests, a shear rate or a shear stress was applied to the ER fluids, and the shear stresses and shear viscosities were observed. The shear stresses and shear viscosities as a function of the shear rate are important behaviors for understanding the rheological characteristics of ER suspensions. Figure 10a,b show the shear stress versus shear rate for the GO/SiO_2_-based ER suspension under different DC electric fields and AC electrical field with a frequency of 1 kHz, respectively. In the absence of an electric field, the shear stresses increase with increasing shear rate without a yield stress, indicating the fluid-like state of the ER suspension. On the other hand, in the presence of an electrical field, the particles form chain-like structures, as shown in Figure 8, and the shear stress curves show Bingham plastic behaviors with obvious yield stress [71]. The field-induced degree of polarization increased with increasing electric field strength, resulting in enhanced electrostatic interactions and more strong chain-like structures. Therefore, the shear stress increases with increasing electric field strength. Under an AC electric field with a frequency of 1 kHz, as shown in Figure 10b, the shear stresses tended to increase progressively with increasing shear rate and deviate to the fitted lines (Bingham model) at a low shear rate range. The dielectric properties of the GO/SiO_2_-based ER suspension showed that the relaxation time of the interfacial interaction was approximately 0.5 kHz, which suggests that the relaxation time of GO/SiO_2_-based ER suspensions was too slow to reach sufficient polarization under high frequency flow, resulting in a relatively low stress value.

Generally, the shear stress behaviors under a DC electrical field can be described using the following Bingham fluid model [72]:

(6) $$\tau =\tau_{0}+\eta_{pl}\dot{\gamma },\;\;\tau \geq \;\tau_{0}$$

(7) $$\dot{\gamma }=0,\;\;\tau <\;\tau_{0}$$

where τ is the shear stress; $\dot{\gamma }$ is the shear rate; $\tau_{0}$ represents the dynamic yield stress; $\eta_{pl}$ reflects the plastic viscosity and generally corresponds to the viscosity at which the shear rate is infinite. The Bingham model divides the shear stress curve into two regions: a low shear rate region, where the shear stress exhibits a flat zone; and at high shear rates, the shear stress increases with increasing shear rate. As shown in Figure 10a, the shear stress curves after the dynamic yield point match the fitted Bingham model well, and the stress in the plateau region is consistent with the dynamic yield stress.

In many ER systems, however, the Bingham fluid model is not sufficient to fit the flow behaviors of ER suspensions. As shown in Figure 10c, the shear stresses of Si-GO-based ER fluids exhibit a reduced region before increasing as the shear rate increased. Therefore, the following six-parameter CCJ model [73] was developed:

(8) $$\tau =\frac{\tau_{0}}{1+{\left(t_{1}\dot{\gamma }\right)}^{\alpha }}+\eta_{\infty }\left(1+\frac{1}{{\left(t_{2}\dot{\gamma }\right)}^{\beta }}\right)\dot{\gamma }$$

where the time parameters, t_1_ and t_2_, combined with the exponents, α and β, control the reduced and rising regions of the shear stress, respectively. The dashed lines and solid lines in Figure 10c correspond to the Bingham and CCJ models, respectively. Obviously, the CCJ model can better describe the shear stress curves. The reduced region may be related to the slow relaxation time (0.05 s) of interfacial polarization, i.e., the slow relaxation time causes the particles to fail to reach a sufficient polarization in time. Hence, the chain-like structures destroyed by shear flow cannot recombine in a timely manner, resulting in reduced shear stress. In addition, the shear viscosity (Figure 10d) increased with increasing electric field strength and showed obvious shear thinning behavior. As the electric field strength increases, the field-induced electrostatic interactions among the particles increased, and the strength of the chain-like structures was enhanced, causing an increase in frictional resistance among particles, which is manifested by an increase in shear viscosity. On the other hand, as the shear rate increases, the chain structures are destroyed gradually, and the shear viscosity decreases gradually, displaying shear thinning behavior.

### 3.4. Dynamic Yield Stress

**Figure 11:**
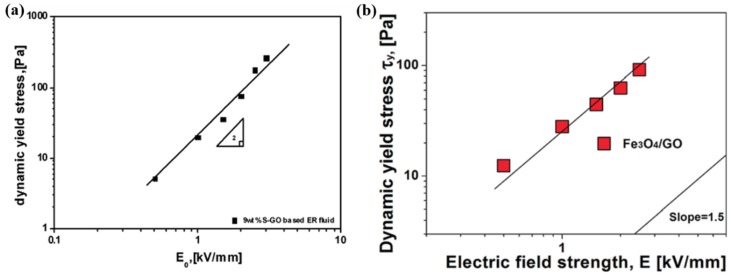
Dynamic yield stress as a function of the electric field strength for (**a**) Si-GO based ER fluid (9 wt.%) [66], (**b**) Fe_3_O_4_/GO based ER fluid (15 wt.%) [55].

The dynamic yield stress is defined as the minimum stress that can break up the chain-like structures continuously, while being recombined continuously by electrostatic interactions. That is, when the shear stress is higher than the yield stress, the particles begin to move with the flow field. The dynamic yield stress is usually estimated by extrapolating the shear stresses near a zero shear rate according to the log-log scaled flow curves. The correlation between the yield stress and electrical field strength is expressed as

(9) $$\tau_{0}\propto E^{m}$$

where E is the electric field strength; m is an exponent usually ranging from 1.0 to 2.0. Among these values, there are two main models. In the first model, when m = 2.0, Equation (9) represents the polarization model [74,75]. Figure 11a shows the dynamic yield stress of the Si-GO-based ER fluid vs. electrical field strength. The fitted line was obtained by $\tau_{0}\propto E^{2.0}$, indicating that the Si-GO-based ER suspension follows the polarization model. In the other model, when m = 1.5, the equation represents a conduction model. As shown in Figure 11b, the Fe_3_O_4_/GO-based ER suspension follows the conduction model [76]. In some cases, however, the exponent does not obey the polarization model or the conduction model. This is because these models are based on monodispersed spherical particles, but the value of the dynamic yield stress is actually related to many factors, such as the particle morphology, size, density, and concentration of the ER fluid.

### 3.5. Response Sensibility

**Figure 12:**
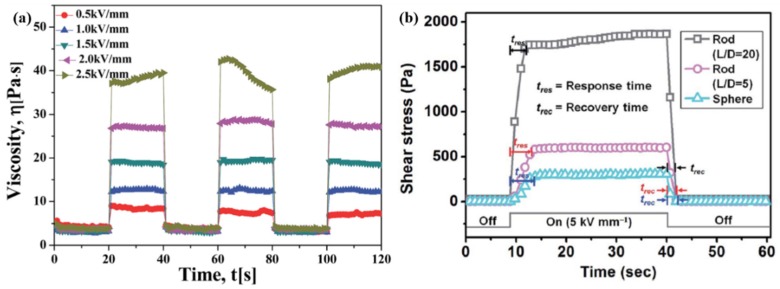
Effect of switching the applied electric field for (**a**) GO-coated silica based ER fluid (15 wt.%) [68], (**b**) series of ER fluids based on GO-coated silica with different aspect ratios [60].

To examine the response sensitivity of the ER suspensions to electrical field stimuli, the flow properties of the ER suspensions were tested while the electrical field was alternately turned on and turned off. Figure 12a shows the change in shear viscosity of GO-coated silica-based ER suspension (15 wt.%) under a square voltage pulse (t = 20 s) at a constant shear rate (1 s^−1^). Under an applied electrical field, the shear viscosity increases instantaneously, and when the electric field is off, the shear viscosity decreases rapidly to an original (0–20 s) zero-field level. This means that the ER fluid is sensitive to the stimuli of the electrical field. Under the stimuli of an electric field, the particles can align rapidly to form chain-like structures, and in the absence of an electrical field, they can return to their freely dispersed state in time. The sensitivity and tunable properties of ER fluids is the main reason for their attention in the industrial applications of smart devices. Figure 12b shows the shear stresses of ER suspensions based on a series of GO-coated silica with different aspect ratios during cyclical switching of the electric field. The response time ($t_{res}$) and recovery time ($t_{rec}$) are defined as the time required to reach 90% of the final shear stress when the electrical field is turned on, and the time required to recover to 90% of the original shear stress when the electric field is turned off, respectively. The ER fluid based on particles with a high aspect ratio exhibited shorter response and recovery times, suggesting that particles with a higher aspect ratio can respond more rapidly to the applied electric field.

### 3.6. Dynamic Oscillation Analysis

In dynamic mechanical analysis, an oscillatory (sinusoidal) deformation (stress or strain) is applied to the ER fluids and the complex modulus can be measured. Phase change in the ER fluid can be also evaluated through the change in storage modulus ($G^{\prime }$) and loss modulus ($G^{\prime \prime }$), and the critical shear strain or frequency value of destroying the chain-like structures can also be obtained, which is critical for evaluating the strength of ER fluids under dynamic shear. When the deformation is small and applied sufficiently slowly, the chain-like structures formed under an applied electric field could be kept without destruction, in which the storage and loss modulus show a stable value; this region is defined as the linear viscoelastic (LVE) region. In this LVE region, the magnitudes of stress and strain are related linearly, which can be represented as $\tau =G^{\prime }\gamma$. Here γ is the strain. Based on this relationship, the elastic yield modulus of the ER fluid under an electric field can be inferred. Therefore, an estimate of the LVE region of the ER fluid and a study of the rheological properties of the region are of great significance.

**Figure 13:**
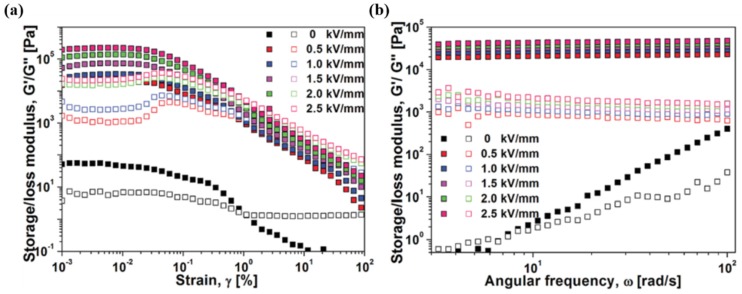
(**a**) Strain amplitude sweep test and (**b**) angular frequency sweep test for Fe_3_O_4_/GO-based ER fluid (15 wt.%) [55].

To observe the linear viscoelastic properties, the strain amplitude sweep test is usually performed at an appropriate frequency. Generally, in the absence of an electrical field, the loss moduli show higher values than the storage moduli, i.e., the viscosity is dominant and the ER suspension exhibits fluid-like properties. In the presence of an electrical field, however, the storage modulus becomes higher than the loss modulus, indicating that the elasticity becomes dominant, and the ER fluid exhibits solid-like properties. When the strain exceeds the critical value of the LVE region, the storage modulus shows obvious collapse, indicating damage to the chain-like structures. As shown in Figure 13a, in the strain amplitude sweep tests of the Fe_3_O_4_/GO-based ER fluid (15 wt.%), the LVE region ranges from 0.001% to 0.02%. Therefore, to avoid damage to the structures by high strain, angular frequency sweep tests (Figure 13b) were carried out at a constant strain of 0.004%. In the absence of an electric field, the moduli increased with increasing angular frequency increases. In contrast, in the absence of an electrical field, they showed a stable value independent of frequency and the storage moduli were higher than the loss moduli. This shows that the elasticity of the ER fluid becomes stronger after the application of an electric field, which is also evidence of the phase change of the ER fluid from a liquid-like to a solid-like phase. In addition, the time-dependent relaxation of the ER fluids can be obtained using the Schwarzl equation [77,78] through the frequency-dependent moduli as follows:

(10) $$G\left(t\right)≃G^{\prime }\left(\omega \right)-0.566G^{\prime \prime }\left(0.5\omega \right)+\;0.203G^{\prime \prime }\left(\omega \right)$$

where G(t) is the relaxation modulus and ω is the angular frequency.

### 3.7. Sedimentation Stability

**Figure 14:**
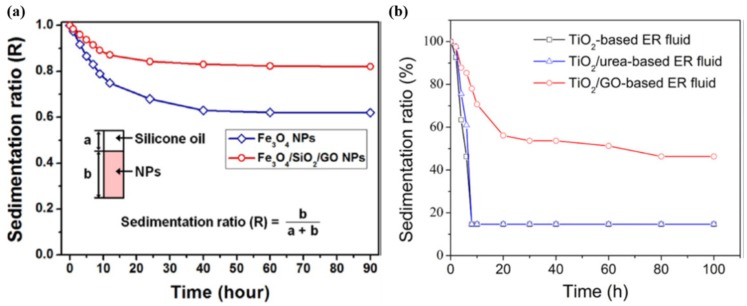
Sedimentation properties of (**a**) Fe_3_O_4_ and Fe_3_O_4_/SiO_2_/GO-based ER fluids (25 vol.%) [64], (**b**) TiO_2_, TiO_2_/urea and TiO_2_/GO-based ER fluids (37 vol.%) [58].

The sedimentation of ER fluids is an important limiting factor, and too severe precipitation will adversely affect its ER performance, making it difficult to use widely in industry. For GO-based ER materials, because GO has a relatively lower density than most of inorganic materials, the density can be reduced when inorganic materials are combined with GO, which can obviously alleviate its sedimentation problem. In addition, GO has good suspension stability in water and many organic solutions, and electrostatic repulsion between oxygen functional groups on the surface of GO can prevent particle aggregation and improve the precipitation stability. As shown in Figure 14a, the Fe_3_O_4_/SiO_2_/GO-based ER fluid shows better sedimentation stability than the Fe_3_O_4_-based ER fluid. Furthermore, TiO_2_/urea and TiO_2_/GO have similar densities of 1.985 and 1.978 g/cm^3^, respectively, but the sedimentation stabilities of an ER fluid based on them are significantly different (Figure 14b). The density of TiO_2_/urea is slightly lower than that of TiO_2_ with a density of 2.047 g/cm^3^, whereas there was almost no difference in the sedimentation stability of the ER suspensions based on them. The obvious improvement of the sedimentation stability for TiO_2_/GO-based ER fluid was attributed to the high surface area of GO; when it was coated on TiO_2_, it behaves as a “parachute” to retard the sedimentation rate of the particles.

To further understand the commonality of GO-based composites and the difference between GO/inorganic and GO/polymer composites, Table 1 lists the main information of the GO/inorganic composites covered in this article, as well as the representative GO/conducting polymer [79,80,81,82], and GO/insulating polymer-based [49,83,84,85,86,87,88] ER materials, including synthesis methods, density (ρ), highest applied electric field strength $E_{max}$, leakage current density at 3 kV/mm, viscosity of off field $\eta_{off}$ at a shear rate of 1000 s^−1^, dynamic yield stress ($\tau_{0}$) at 3 kV/mm, slope of the yield stress depending on the electric field strength, storage/loss modulus (G′/G″) at 0 and 3 kV/mm, interfacial polarizability (Δε), and relaxation time (λ) of interfacial polarization.

The synthesis of GO/inorganic composites utilizes electrostatic interactions, in-situ growth of inorganic component on GO, and chemical grafting methods. The latter two methods are believed to strengthen the connection between the two components because of the formation of chemical bonds, while the synthesis using electrostatic interaction is preferred because it is easy to operate and has high efficiency. In comparison, the recently developed synthesis method involves the addition of GO/polymer composites to the usual in-situ polymerization [79,80,81] and chemical grafting [82] methods, Pickering emulsion polymerization [83,84,85], and surface initiated atom transfer radical polymerization (SI-ATRP) [86,87,88,89] methods. Among them, the in-situ polymerization method is generally used for the synthesis of GO/conducting polymer composites because the monomer of a conducting polymer can attach to GO by π-π stacking. The synthesis route of Pickering emulsion polymerization is simple and uniform spherical composites can usually be obtained, in which the hydrophilic GO acts as a solid stabilizer for the hydrophobic monomers in the water phase. The important feature of SI-ATRP is the ability to control the reduction of GO while synthesizing composites, thereby controlling the materials to have the desired electrical conductivity to contribute to stronger ER effects.

In general, the morphology of the composite material is divided into two main types: spherical and sheet. In addition, in-situ synthesis and SI-ATRP usually only obtain sheet-like composite materials based on GO sheets, whereas Pickering emulsion polymerization usually obtains spherical GO-coated polymer composites. On the other hand, the morphology of GO/inorganic composites synthesized by electrostatic interactions can be designed more easily, such as GO-wrapped silica rods [59] with different aspect ratios. Higher Δε, shorter λ and higher yield stress were obtained with increasing aspect ratio of rods.

The density (ρ) of composites not only has an important influence on the sedimentation of ER fluids because their sedimentation problem is an important factor limiting its ER performance, making it difficult to use widely in industry, but also because it affects the ER properties. GO-coated silica spheres [58] with different densities show that as the density decreases, the sedimentation stability of the ER fluid increases, Δε increases, λ becomes shorter, and the dynamic yield stress at 3 kV/mm increases. As this point, enhancement of the ER effect has been attributed to the particles with a low-density being more mobile. Therefore, the particles are more sensitive to electric field stimuli. GO/inorganic composites have a lower density of GO and a special thin sheet morphology can be used as a “parachute” to improve the sedimentation stability. On the other hand, the insertion of a non-polar polymer composed of GO/polymer composites has been shown to result in better sedimentation stability because of the relatively poor compatibility between polar GO and non-polar silicone oil [49,86]. Furthermore, Mrlik et al. [87,88] reported that the longer the polymer chain, the higher the stability, which may be because these particles are more lyophilic, causing their repulsion, and eliminating particle aggregation.

According to the slope of the yield stress vs. the electric field strength, most of them are close to 1.5, which conforms to the conduction model. In addition, some follow the polarization model with a slope of 2.0, or the polarization model converts to a conductive model at high electric field strengths. A few materials exhibit a low slope of 1.0 or 1.5 at low electric field strengths and change to 1.0 at high electric field strengths. Moreover, the common feature of these materials is the lower electrical conductivity of particles ($\sigma_{p}$). Therefore, a too low $\sigma_{p}$ (>10^−12^ S/cm) may lead to unsatisfactory ER performance.

The storage modulus (G’) and loss modulus (G″) are also important data for measuring the viscoelasticity of ER fluids. In the absence of an electric field, G′ shows a lower or slightly higher value than G″, indicating that the ER fluids are in a liquid-like or weak solid-like state. Under an electric field strength of 3 kV/mm, however, G′ is an order of magnitude higher than G″, meaning that elasticity predominates and the ER fluids exhibit stronger solid-like properties.

In addition, the insertion of inorganic components into the composite materials of GO to combine the dielectric properties of the inorganic component with the appropriate conductivity of GO can result in stronger ER characteristics compared to GO/polymer composites. GO/inorganic composite materials usually have higher thermal stability, abrasion resistance, and corrosion resistance. Table 1 shows that the shape, size and density of the GO/inorganic composite materials are easier to design than the GO/polymer composites, which is advantageous for designing different characteristics of the ER material in different applications.

Although many studies on the ER properties have been performed under DC electric fields, the behavior of ER fluids under AC electric fields has also been reported. The frequency ($f_{m}$) at which the loss factor of the ER fluid reaches its maximum value can be obtained from the dependence of the dielectric properties of the ER fluid on the frequency of the electric field. When the frequency of the applied electric field is lower than $f_{m}$, the ER fluid is sufficient to produce ER characteristics similar to those under a direct current electric field. On the other hand, when the frequency is higher than $f_{m}$, an unsatisfactory ER effect will be exhibited because the dielectric relaxation of the ER fluid falls behind the electric field frequency. In addition, as the frequency of the electric field increases, the ER effect continues to decrease.

On the other hand, a high leakage current density (J) limits the range of electric field strengths that can be applied to ER fluids as a limiting factor in the applicable electric field strength because J increases with increasing electric field strength [55], and tends to cause short circuits and safety problems under high electric field strengths. The value of J at 3 kV/mm shown in Table 1 does not exceed 10 μA/cm^2^, but the amount of related information is insufficient. On the other hand, the leakage current density is closely related to the electrical conductivity of the particles ($\sigma_{p}$). Therefore, adjusting $\sigma_{p}$ is an important means of controlling the leakage current density. From the table, materials with $\sigma_{p}$ of 10^−6^ to 10^−13^ can be applied at an electric field strength of at least 2.0 kV/mm. When an electric field is applied, the particles are arranged in chain-like structures between the electrodes, which may cause an excessive leakage current density, resulting in a safety problem. When the shear rate increases, the chain structures are deformed or destroyed, and the leakage current density is correspondingly reduced. As the electric field strength continues to increase, however, more and stronger chain structures are formed, resulting in a higher leakage current density. Moreover, a stable chain structure may result in a high leakage current density at higher shear rates, thereby increasing the risk of security problems. To reduce this risk, it is important to adjust the electrical conductivity of the particles. In this paper, particles with electrical conductivity of 10^−6^ S/cm or lower were shown to act as safe ER materials. In addition, the leakage current density may increase sharply with increasing operating temperature [90], which limits the application of ER fluids in high temperature environments.

Furthermore, there are many factors affecting the characteristics of ER fluids, such as the shape, size, conductivity of the particles, and the particle concentration of the ER fluid. In industrial applications, ER fluids with a high yield stress are usually required. On the other hand, excellent sedimentation stability and low leakage current density are important conditions for ER fluid to maintain long-term effects under high electric field strengths. In addition, with the advent of the intelligent era, the controlled rheological properties of ER fluids make it possible to create more applications in smart devices. In this case, the sensitivity of an ER fluid to electric field stimulation and the ability to recover when the electric field is removed are particularly important.

## 4. Conclusions

This paper reviewed various GO/inorganic composites adopted in ER fluids, focusing on the fabrication mechanisms and ER properties, including the dielectric properties and sedimentation stabilities. The synthesis mechanism of the GO/inorganic composites is divided into two main categories. One is to separately synthesize GO sheets and inorganic particles, and then use electrostatic or chemical grafting to fabricate the composite material. The other is to synthesize inorganic materials directly on GO sheets, in which case, it is generally subjected to in-situ hydrolysis of the inorganic particle precursor. The numerous oxygen functional groups on the surface of GO provide important convenience for synthesis.

The dielectric characteristics of GO/inorganic ER materials are strongly related to the ER effect, in which materials possessing a high permittivity difference between zero and infinite frequency and a rapid polarization rate can contribute to stronger ER effects. The morphology of ER particles is also an important factor on the ER effect. GO/inorganic composite particles with a higher aspect ratio contributed to a larger electric field-induced dipole moment, resulting in better polarization. The higher the aspect ratio of the rods, the more inclined they are to obtain higher polarizability and shorter relaxation time of interfacial polarization. In addition, particles with a low density and better dielectric properties including GO composites could contribute a high mobility, such that they have a low response time, thereby contributing to better ER properties.

The sedimentation stability of GO/inorganic composite-based ER fluids is also due mainly to the properties of GO. First, the oxygen-containing functional groups on the surface of GO allow them to be well dispersed in water as well as in common organic solvents, and electrostatic repulsion between these functional groups prevent aggregation, which reduces the rate of sedimentation. Second, GO can reduce the overall density of the composites and reduce the density difference with the carrier liquid, thereby reducing settling. In addition, the attached GO acts as a “parachute” to slow down the sedimentation of composites owing to its sheet morphology and high specific surface area.

As can be determined from a comprehensive summary of currently known GO/inorganic composite ER materials we covered in this review, some materials can not only hold yield stresses of up to several thousand Pa, but also exhibit reasonable sedimentation stability. Moreover, the relatively good thermal stability, wear resistance, and corrosion resistance of GO/inorganic composites add to the market for its application in the industry. In addition, compared to GO/polymer composites, GO/inorganic composites have powerful advantages that can be designed, including the shape, size, and density, which assists in the design of suitable materials for a range of applications. Overall, GO/inorganic composites will benefit from more in-depth research.

## Figures and Tables

**Table 1:** Main information of GO-based composites applied to ER fluids.

Materials	Synthesis Method	Morphology	ρ (g/cm^3^)	$\sigma_{p}\;(S/\mathrm{cm})$	Particle Concent-Ration	$E_{max}\;(\mathrm{kV}/\mathrm{mm})$	J	$\eta_{off}\;(\mathrm{Pa}\cdot s)$	$\tau_{0}\;\mathrm{at}\;3\;\mathrm{kV}/\mathrm{mm}\;(\mathrm{Pa})$	Slope	$G^{\prime }$$/G^{\prime \prime }$ (Pa)	$G^{\prime }$ $/G^{\prime \prime }$	Δε	λ (s)	Sedimen-Tation Ratio
GO [43]	Modified Hummers method	Sheet	1.78	6.02 × 10^−6^	5 wt.%	2.5	_	~0.1	_	_	_	_	0.45	1.3 × 10^−5^	_
GO/TiO_2_ [53]	Electrostatic interaction	Sheet	_	5.5 × 10^−10^	15 wt.%	3.0	_	_	160	_	_	_	_	_	_
GO-wrapped titania [56]		Sphere	_	_	10 vol.%	3.0	1	~0.2	600	1.47	~30/90	~2 × 10^5^ 2 × 10^4^	2.7	5 × 10^−3^	45%
TiO_2_/GO [58]		Sphere	1.978	_	37 vol.%	5.0	<10	_	~8 × 10^3^	_	_	_	_	_	50%
Fe_3_O_4_/GO [55]		Sphere	_	10^−7^	15 wt.%	3.0	_	~0.2	100	1.5	~50/10	~2 × 10^5^ 2 × 10^4^	_	_	_
Fe_3_O_4_//SiO_2_/GO [65]		Sphere	_	_	25 vol.%	3.0	_	~0.08	15	_	_	_	_	_	82%
GO/Al_2_O_3_ [54]		Sphere	_	1.24 × 10^−13^	20 wt.%	5.0	_	~0.07	92	1.0	_	~3 × 10^4^ 3 × 10^3^	_	_	_
GO-wrapped silica [60]		Sphere	_	_	5 wt.%	5.0	_	_	~100	2.0–1.5	_	_	0.71	1.592	_
		Rod (1:5)	_	_	5 wt.%	5.0	_	_	~250	2.0–1.5	_	_	1.4	1.007	_
		Rod (1:20)	_	_	5 wt.%	5.0	_	_	~800	2.0–1.5	_	_	2.78	0.634	_
GO-coated silica spheres [59]		GO/nSiO_2_	2.67	_	3 wt.%	4.0	_	_	9.88	_	_	_	0.94	1	65%
		GO/p1SiO_2_	2.52	_	3 wt.%	4.0	_	_	17.08	_	_	_	1.94	0.64	60%
		GO/p2SiO_2_	2.28	_	3 wt.%	4.0	_	_	20.43	_	_	_	2.12	0.64	50%
		GO/epSiO_2_	2.03	_	3 wt.%	4.0	_	_	30.18	_	_	_	2.65	0.4	45%
GO/SiO_2_ [67]		Sheet	_	_	3 vol.%	2.0	_	_	_	_	_	_	0.9	4.5 × 10^−7^	_
Si-GO [66]	In-situ growth	Sheet	_	_	9 wt.%	3.0	_	~0.05	264	2	_	~5 × 10^4^ 5 × 10^3^	2.3	5 × 10^−2^	_
GO-Si [68]	Chemical grafting	Sphere	2.003	_	15 wt.%	2.5	_	~0.05	_	1.5	~30/10	_	0.06	6 × 10^−6^	_
GO/PANI [79]	In-situ polymerization	Sheet	_	_	10 vol.%	2.5	_	~4	_	2.0–1.5	_	_	_	_	_
GO/NCOPA [80]		Sheet	_	_	10 wt.%	5.0	_	~0.1	~200	2.0–1.5	~7 × 10^3^ 2 × 10^2^	_	1.98	1 × 10^−3^	_
GO/P2MAN [81]		Sheet	1.43	_	10 vol.%	2.0	_	~0.1	_	1.5	~0.5/2	_	_	_	~85%
PPDA/GO [82]		Sheet	_	_	8 wt.%	2.5	_	~10^3^	_	_	_	_	1.71	_	_
POSS-GO [49]	Chemical grafting	Sheet	_	_	~3 wt.%	3.0	<0.5	~0.7	~600	1.53	_	~5.8 × 10^3^ 1 × 10^3^	_	_	_
PS/GO [83]	Pickering emulsion polymerization	Sphere	_	_	10 vol.%	2.0	_	_	_	1.5	~20/200	_	2.54	2.54 × 10^−4^	_
PMMA/GO [84]		Sphere	1.27	-	10 vol.%	2.5	_	_	_	1.5	~4/5	_	0.51	5 × 10^−3^	_
PGMA/GO [85]		Sphere	1.41	8.7 × 10^−12^	10 vol.%	2.0	_	~0.07	_	1.5–1.0	~1/1	_	2.6	2.6 × 10^−3^	_
GO-PGMA [86]	SI-ATRP	Sheet	_	6.17 × 10^−7^	10 wt.%	2.0	_	_	_	1.46	_	_	1.81	9 × 10^−3^	90%
GO-PBMA [87]	SI-ATRP	Sheet	2.21	6 × 10^−7^	5 wt.%	2.5	_	_	_	1.48	_	_	1.37	5 × 10^−3^	60%
GO-PHEMATMS [88]	SI-ATRP	Sheet	_	6 × 10^−6^	5 wt.%	2.5	_	_	_	1.44	_	_	2.11	2 × 10^−3^	90%
